# Videoconferencing Psychotherapy During the Pandemic: Exceptional Times With Enduring Effects?

**DOI:** 10.3389/fpsyg.2021.589536

**Published:** 2021-02-19

**Authors:** Javier Fernández-Álvarez, Héctor Fernández-Álvarez

**Affiliations:** ^1^Department of Basic Psychology, Clinical and Psychobiology, Jaume I University, Castellón de la Plana, Spain; ^2^Asociación Aiglé, Valencia, Spain; ^3^Fundación Aiglé, Buenos Aires, Argentina

**Keywords:** COVID-19, psychotherapy, psychotherapists, telehealth, videoconferencing psychotherapy, telemental health, e-mental health

## Abstract

With the advent of COVID-19, a sudden, unexpected, and forced shift has been produced in the field of psychotherapy. Worldwide, many therapists closed their offices and started to deliver psychotherapy online through a screen. Although different media started to be incorporated, videoconferencing is undoubtedly the most common way in which therapists are doing therapy these days. This is catalyzing a rapid change in the practice of psychotherapy with probable lasting effects and deserves to be carefully reflected upon. Therefore, in this paper our aim is to outline the main challenges for a medium that may have arrived to stay. In that sense, we review the literature to describe the state-of-the-art regarding the main aspects of videoconferencing psychotherapy as well as to suggest possible avenues for future research and practice.

## Introduction

Although no consensus exists among experts regarding what comprises Internet-delivered interventions (Smoktunowicz et al., 2020), there is no doubt that they have gained a central role in the clinical psychology realm. A large body of evidence supports the incorporation of different technologies, with different media and degrees of human support (Andersson et al., 2019). Even psychotherapy, which has historically involved an in-person shared space, has slowly but gradually incorporated more and more use of technologies. Fundamentally, the application of videoconferences in routine practice has been progressive and is mainly explained by practical reasons, such as geographical barriers, treatment-seeking stigma, or flexibility in scheduling sessions (Nickelson, 1998; Backhaus et al., 2012). Research on the remote delivery of therapy has increased along with this increasing use, and accordingly, a large body of evidence has been produced during the last two decades showing the efficacy of delivering psychotherapy through videoconference, even with comparable results to in-person therapy (Varker et al., 2018; Batastini et al., 2020). However, its application in routine practice has not been widespread, with almost all therapists having no experience.

With the advent of the coronavirus disease (COVID-19), a sudden, unexpected, and forced shift has been produced. Worldwide, many therapists closed their offices and started to deliver psychotherapy online. The use of technology became the only way in many countries to provide psychotherapy, and an overnight transition from in-office to online practice occurred. Given that videoconferencing constitutes a similar way of delivering therapy to traditional in-person psychotherapy, it has been rapidly incorporated (Sammons et al., 2020; Wind et al., 2020).

Although this massive dissemination is positive since millions of people could potentially benefit from these treatments, a series of questions remain unanswered. In this paper, we aim to outline the main challenges for a modality that may have arrived to stay.

## Issues in Working With Videoconferencing

### General Therapeutic Targets

The use of videoconferencing psychotherapy (VCP) does not change the needs of patients and thus the general therapeutic goals. Patients’ specific demands may have changed due to the pandemic, but their suffering will still be centered around their difficulties with the two main components of dysfunction: self-dysfunction and interpersonal dysfunction (Hopwood et al., 2013). Besides, it is important to focus the work on the two dimensions in which the psychophysiological functioning of the organism is deployed: behavior and experience.

Demanding scenarios such as lockdown or uncertainty about the aftermath of the pandemic constitute stressors that may particularly affect people who already had maladaptive strategies for coping with reality. In many cases, the context merits exacerbated dysfunctional reactions regarding our mental health. However, it is key to keep in mind that although the context operates as a fundamental variable in peoples’ lives, the core aspects regarding the ways of organizing experience are personality and its components, such as schemas, attachment styles, regulatory capacities, and interpersonal functioning, among others (Livesley, 2012).

It is necessary to think outside the box and not just consider the dangers and negative aspects of the pandemic. The current context obliges us to live under constant threat, and therefore, the situation reminds us that we are fragile beings (Wong, 2020). Implementing VCP, in particular due to a forced situation like COVID-19, may be an opportunity to work on issues that otherwise would not have been possible to address. The presence of a difficult situation may facilitate the setting of new goals. That is, this context may also foster the promotion of positive changes such as meaning in life as an important therapeutic target (Hill et al., 2017). Meaning in life has proven to be a very powerful way of regulating emotions as well as promoting positive psychology principles that, far from focusing on the positive as a superfluous thing, considers existential sorrow to be a way to find freedom (Wong, 2011). The unexpected consequences of the pandemic foster a discussion that is more important than ever: What should we pursue in life? Are we living according to our values?

### Therapeutic Alliance

There are substantial differences between in-person psychotherapy and VCP that may have an impact on how the therapeutic alliance is developed. For instance, in VCP, both patients and therapists have the possibility of having feedback from their own cameras. Certain patients and therapists (e.g., narcissistic or socially anxious individuals) may pay too much attention to their own behavior, and this may be detrimental to therapeutic communication (Payne et al., 2020). Moreover, in-person psychotherapy uses a physical shared space, entailing the immediacy of the sensory experience and thus an undoubtedly qualitatively different exchange. The most evident difference between in-person psychotherapy and VCP is the potential technical difficulties that may arise during the latter. As explained by Markowitz (2020), an unstable connection, a frozen screen, delayed audio or poor lighting are some of the difficulties that may impair engagement in therapy. Additionally, as described by Thompson-de Benoit and Kramer (2020), direct eye contact, tone of voice, the ability to have an open posture, body movements, synchrony, and attunement are some of the communicative channels that may be hampered in VCP. That is, the paralinguistic, non-verbal and prosodic aspects of communication may be affected. Principles that ground embodied cognition enable one to grasp how physicality is key for information processing, involving bodily aspects that may not be transferable to remote modalities (Caramazza et al., 2014). A stooped posture, a shaking leg or a clenched fist are invisible in VCP. That relevant information is missed in VCP both for therapists and patients.

Not taking into account these differences that exist between modalities may affect the development of the therapeutic process and, consequently, result in early dropouts. Other ruptures in remote psychotherapy may be exacerbated due to the aforementioned technical problems or disappointment with the restricted possibilities that this modality permits. Identifying both confrontational and withdrawal ruptures and implementing techniques to resolve them is crucial, and there are initial suggestions regarding how to deal with this issue in VCP (Dolev-Amit et al., 2020).

Beyond the conceptual debate around the establishment of therapeutic alliance in VCP, a growing body of evidence shows that it can be established, presumably with comparable results to in-person psychotherapy (Simpson and Reid, 2014; Norwood et al., 2018; Lopez et al., 2019). The results of these studies converge on the conclusion that a therapeutic alliance can be successfully formed in VCP. Indeed, Lopez et al. (2019) conclude that VCP “*…is a viable modality with the potential to improve access to care with a low impact on therapeutic alliance*.” The authors suggest that the therapeutic alliance is not particularly affected, and therefore, it does not hinder any therapeutic progress. Although therapeutic alliance can be well established in VCP, it is premature to conclude that it is equal to in-person psychotherapy.

Undoubtedly, the therapeutic alliance constitutes a core element in all psychological treatments (Flückiger et al., 2018). Indeed, the therapeutic alliance may be conceived as a moderator or an active mechanism of change (Zilcha-Mano, 2017; Baier et al., 2020). The longstanding tradition of therapeutic alliance research in in-person psychotherapy has produced several lines of research that have provided profound insight into how it is deployed (Norcross and Lambert, 2019). However, there is little research thus far on the role of the therapeutic alliance in treatment in VCP research compared to in-person psychotherapy. One topic it would be relevant to conduct research on in VCP research is the reciprocal dependency between the therapeutic alliance and symptomatology, as there is mounting evidence in research on in-person treatment, which suggests that the formation of a strong therapeutic alliance precedes symptomatic change (Zilcha-Mano et al., 2014; Zilcha-Mano, 2017). For now, there are only a few examples of VCP research on this issue, without the same complexity as research on in-person psychotherapy (Bouchard et al., 2020).

From a neurobiological point of view, the attachment bond is usually associated with the 9-amino-acid cyclic neuropeptide oxytocin (Schneiderman et al., 2014), which in turn is a marker of the therapeutic alliance and alliance ruptures (Zilcha-Mano et al., 2018, 2020). Additionally, based on the Polyvagal Theory (Porges, 2007), there is research showing that higher in-session heart rate variability (specifically the high-frequency power) facilitates the establishment of therapeutic alliance, and this predicts symptomatic improvement (Blanck et al., 2019). It will be important to demonstrate that these associations occur in remote modalities as well. Should research be conducted on these more nuanced aspects, it would not be surprising to find that differences between in-person and remote psychotherapy emerge concerning the quality of the therapeutic relationship.

There is a great difference regarding the establishment of the therapeutic alliance between treatments that begun with an in-person modality and transitioned to VCP and treatments that were delivered remotely from the beginning. In treatments that make a transition to VCP, it is important to consider the necessity of making a new contract (Inchausti et al., 2020). Beyond the bond, the classical conceptualization of the therapeutic alliance entails objectives and tasks. Even though the bond may be very strong, the tasks and specific goals previously agreed upon should be closely examined to determine whether it is necessary to introduce changes given the new circumstances. Concerning specific objectives, there may be some nuances, but overall, they are also transferable from in-person to VCP. The greatest difference between in-person and VCP may lie in the tasks. Due to either the modality or the context, the usual tasks cannot be conducted. Commonly used techniques in in-person psychotherapy may need a process of adaptation to be implemented in VCP. An illustrative example is the delivery of tele-chairwork (Pugh et al., 2020).

On a positive note, it has been found that VCP can promote more disinhibition and openness due to the possibility of producing a sense of safety and a more neutral power balance. At the beginning of treatment, this neutral disposition in the bond can foster greater disclosure among patients who have certain interpersonal patterns (e.g., submissive patients) and may benefit from a less confrontational relationship (Simpson et al., 2020).

### Adapting the Interventions to the Patients’ Preferences, Characteristics, and Clinical Problems

Evidence-based practice in psychology entails the integration of best available research, clinical expertise, and patient preferences and values (APA Presidential Task Force, 2006). Taking into account the preferences and values permits to adapt the treatment to each individual. Cultural sensitivity emerges more than ever as an essential aspect to consider, given that there are substantial differences depending on a range of factors for the practice of VCP during critical times such as the current COVID-19 pandemic. Therefore, it is relevant to tailor the treatment according to the following aspects:

#### Clinical variables such as psychopathological severity

It is still very important to assess suicide risk, in particular in the context of disasters like COVID-19 in which suicide rates are expected to increase (Gunnell et al., 2020). It is crucial to adopt emergency measures if suicidal thoughts or attempts are detected (Gilmore and Ward-Ciesielski, 2019; Jobes et al., 2020). For serious mental illness as well as for particular clinical groups that may be hindered from working properly through videoconferencing, specific guidelines should be elaborated and followed.

There are certain clinical situations that may be more challenging than others. For instance, dealing with a person with a severe eating disorder entails obtaining session weights or having family meals, which demands specific solutions for working remotely (Matheson et al., 2020). Likewise, the procedure for conducting exposure therapy may be drastically changed. An exposure task for social anxiety disorder in remote psychotherapy can be adapted by including unknown people in a videoconference call (OxCADAT, n.d.). Numerous papers have been published for treating clinical conditions *via* remote therapy, including obsessive compulsive disorder (McKay et al., 2020), bipolar disorders (De Siqueira et al., 2020), suicide (Mcginn et al., 2019), psychosis (DeLuca et al., 2020; Hasson-Ohayon and Lysaker, 2020), post-traumatic stress disorder (Aafjes-van Doorn et al., 2020b; Fina et al., 2020), sleep disorders (Arnedt et al., 2020), among others.

#### Sociodemographic variables

The socioeconomic background or digital literacy should be particularly taken into consideration before starting a VCP treatment (Nelson et al., 2017; Markowitz et al., 2020). That means that the therapist needs to design the specific goals and tasks in accordance with the patient’s characteristics, needs, and preferences. This is particularly true given that, worldwide, people who suffer the most are vulnerable and underserved populations (Frankham et al., 2020). The present situation involving the presence of the COVID-19 pandemic is not exceptional in this regard. Socioeconomically excluded people or people at high risk such as elderly people are logically those who potentially would need more help under these circumstances, but paradoxically also have less access to psychotherapy, including to VCP.

#### Acceptance and attitudes toward technology

Although it was thought that patients were resistant to VCP in its early days, research shows that overall patients have a positive attitude toward VCP (Trondsen et al., 2014; Bleyel et al., 2020). Hence, it is essential to consider the experience of the patients with technology as well as with previous psychological treatments and, accordingly, to determine the extent to which the patients consider that such a treatment may be beneficial for them. Patients’ resistance may be explored and potentially coped with motivational interviewing strategies (Walker et al., 2020).

### Adapting the Interventions to Different Modalities and Settings

VCP was first delivered fundamentally in individual formats, for adults and in private practice. Recently, as a consequence of the need for rapidly adapting practice to remote delivery, VCP has extended to all formats (family, couple, and group therapy formats), populations (children, adolescents and elder people) and settings (e.g., hospitals, university counseling centers, community clinics, prisons).

Family therapy is particularly necessary for certain clinical situations (Amorin-Woods et al., 2020), such as those that affected younger people and adolescents (Burgoyne and Cohn, 2020). An illustrative example is the work with patients having an eating disorder (Matheson et al., 2020) or cases involving child maltreatment (Racine et al., 2020). Couple therapy has been in increasing demand recently, due to the significant rise of conflicts that emerge as a consequence of the adverse aftermath of confinement and the pandemic (Lebow, 2020; Sahebi, 2020; Stanley and Markman, 2020).

A variety of circumstances affect the usual functioning of group therapy, but the preliminary evidence suggests that efficacy has been similar to that observed previously (Marmarosh et al., 2020). There is also evidence that group VCP allows for the development of cohesion to a similar extent as in in-person group psychotherapy (Gentry et al., 2018; Lopez et al., 2020). Among the barriers, the participation of several patients in VCP may reduce the communication fluency of the group and hinder the usual dynamics (Weinberg and Rolnick, 2019). Working with groups necessarily increases the number of interactions and, accordingly, the complexity of any system such as therapeutic groups (Aureli and Schino, 2019). If, normally, group therapists have to have a higher degree of attentional flexibility and more diverse intervention procedures than individual therapists in VCP, this is particularly relevant.

### The Person of the Therapist

There are still a lot of unknown aspects, but it is an undoubted global phenomenon that VCP became an essential tool regardless of therapists’ therapeutic orientation, the clinical conditions, and even the therapists’ previous experience with technology (Humer et al., 2020b; Sammons et al., 2020). Besides, several studies (e.g., Békés and Aafjes-van Doorn, 2020; Humer et al., 2020a; Jurcik et al., 2020) have demonstrated that since the massive incorporation of videoconferencing, therapists’ attitudes toward it have improved.

Psychotherapists would greatly benefit from developing a self-reflective attitude during the whole process of therapeutic alliance building in remote psychotherapy as well as other aspects that may hamper (and potentiate) the therapeutic work. Under these exceptional circumstances occurring during the COVID-19 pandemic, people and therapists all over the world are not the exception, had their routines disrupted and their sense of wellbeing challenged. For the first time, many therapists may be overwhelmed by the same complaints and problems as their patients (Hasson-Ohayon and Lysaker, 2020). Besides, in many cases, the caseload of patients has been reduced, impacting their income (Sammons et al., 2020). Moreover, therapists are not particularly prepared for this kind of modality, and therefore, initial evidence suggests that therapists find it more wearying to do VCP, probably as a consequence of the aforementioned reduced channels of communication (Hoffmann et al., 2020). Likewise, therapists inexperienced with VCP have higher levels of self-doubt and anxiety and feel less competent and confident about their professional skills (Aafjes-van Doorn et al., 2020a).

It has been demonstrated that the adoption of VCP depends a great deal on the attitudes of the providers, including psychotherapists. In a systematic review of 38 studies, it has been found that previous experience with VCP is highly related to having positive attitudes toward it. Besides, therapists’ satisfaction levels with VCP are overall high throughout the studies, although the samples do not represent all psychotherapists (Connolly et al., 2020).

All these aspects necessarily entail an unusual professional and emotional impact. Indeed, ample evidence has recently emerged showing that in COVID-19 times, health professionals are prone to suffer, not only due to the same stresses as everyone else but also due to the necessity of responding to the contextual demands of working in the health care system in such an unusually stressful time (Braquehais et al., 2020). However, mental health professionals working remotely may also have a great burden. Hence, self-care practices that psychotherapists can adopt are essential (Hoffman, 2020).

### Supervision and Training

Fortunately, in recent years, online supervision has become practiced and studied more often, leading to a set of recommendations regarding how to best implement it (Rousmaniere et al., 2014). Just like the work with patients, videoconferencing supervision is more flexible in terms of scheduling meetings, which can be especially important in critical situations. The potential difficulties that may arise in videoconferencing supervision can be counteracted with a clear framework at the time of development of the supervisory alliance. In that sense, it is relevant to consider possible variations in the alliance, which is a matter of importance just as between patients and therapists (Watkins, 2014). The principles that govern group therapy should also be applied to group supervision. Both peer and traditional supervision could be taken as a first step toward the training process of psychotherapists doing VCP.

According to trainees receiving online supervision, it is a valuable component for the training process (Bernhard and Camins, 2020). Indeed, online supervision may serve as a first step toward the establishment of structured training programs. Actually, given the massive dissemination of VCP, it is urgent that psychotherapists be trained to incorporate VCP efficiently into their routine practice. So far, there are a few existing studies of VCP training programs (Colbow, 2013; McCord et al., 2015; Dopp et al., 2017; Perle, 2020), and despite the undoubted attention that has been recently given to the topic due to the onset of the pandemic, there is still a dearth of systematic knowledge regarding VCP training (Hames et al., 2020). Until now, it seems a mere intuitive transition from traditional in-person training.

Training programs should be based on the evidence-based principles that have been shown to enhance therapeutic effects, such as deliberate practice (Prado-Abril et al., 2018). The valuable progress that has been made in in-person psychotherapy should be applied to VCP. In this sense, it is important to avoid disseminating manualized treatments and instead train therapists in general principles of change (Castonguay and Beutler, 2006; Castonguay et al., 2019; Goldfried, 2019; Boswell et al., 2020). It is important to avoid incurring the infructuous dispute between specific therapeutic schools and focus the efforts on achieving therapeutic competence (Cooper et al., 2019). There were already examples of VCP guidelines even before the outbreak of the pandemic (Yellowlees et al., 2010; Turvey et al., 2013; McCord et al., 2020; Smith et al., 2020), but it is expected that the mounting evidence that is being produced and disseminated due to the pandemic will yield valuable insights regarding how best to practice VCP.

### Ethical Considerations

These days, ethical considerations are usually reduced to the privacy dimension. That includes informed consent from patients doing VCP, the security of the platforms, and the guarantee that any stored data will be treated according to data protection regulations, among other aspects. However, ethical issues also include accounting for the safety of the patients, competence of the therapists, legal issues regarding the regulation of the practice, consultants’ autonomy, and commercial contracts (in particular for liberal and third parties’ professionals), among other issues (Lustgarten and Elhai, 2018; Stoll et al., 2020a,b).

## Concluding Remarks

Certainly, remote human interaction will increase in the coming years. This has already been happening for at least a decade. Yet, the outbreak of the pandemic has notably accelerated this process. Psychotherapy will definitely not be the exception to the rule, and therefore, it is crucial to outline how the field will be transformed in the near and long term. Most probably, the implementation of VCP psychotherapy will increase in the next few years (Norcross et al., 2013), and this will happen in a context of the decline in the consumption of psychotherapy (Gaudiano and Miller, 2013). Therefore, we should guarantee the highest standards to differentiate psychotherapy from pseudoscientific disciplines and to demonstrate the value of incorporating psychotherapy into the ever-growing pharmacological treatments.

While it may be true that preliminary research comparing in-person therapy to VCP yields comparable results in terms of efficacy, it would be inaccurate to conclude that both approaches have similar empirical support. Despite presenting promising results, VCP is only in its beginning as a research field. Thus, research and training are key for the advancement of VCP, and this scenario should be taken as an opportunity to foster also the advancement of the field of psychotherapy.

Real world implementation of evidence-based principles would mean strengthening the active collaboration between researchers and practitioners, redounding to the proliferation of practice research networks in which the practice is evidence based and the evidence is practice based. That would mean a reciprocal enrichment both for practitioners and researchers (Castonguay et al., 2015). However, this context undoubtedly facilitates the possibility of improving the attitudes of therapists and consultants toward VCP and by extension toward other technological tools (Wind et al., 2020). Accordingly, a brighter future can be expected if more collaborative research in naturalistic settings occurs.

On a relative but different note, it is important to reflect on the role that VCP will have in the future of psychotherapy. That is, for many psychotherapists, the use of remote modalities constitutes a suboptimal resource that is necessary in order to continue their work. However, many stakeholders consider this an efficient way of increasing the prevalence of mental health treatment. Although many therapists may indeed prefer this modality, and for a range of mild conditions, it is proving to be equally efficacious, the possibilities of in-person therapy seem to still be superior.

Indeed, there are stakeholders that are advocating for the incorporation of completely self-applied online interventions with minimal contact. In fact, the evidence is conclusive regarding the usefulness of low-intensity treatments mainly through Internet interventions to improve access to treatment of common mental disorders (Andersson et al., 2019). In that sense, it is timely to review the paper by Barlow (2004) in which he differentiated psychological treatments from psychotherapy. Briefly put, for public concerns and to diminish the massive clinical manifestations related to mental health, all evidence-based psychological treatments may be of importance, including brief protocolized procedures. However, psychotherapy is only one of the possible psychological treatments and most often differs from other psychological treatments in the sense that the main objective is not only symptomatic reduction but also the reorganization of the personal system and the improvement of the quality of life. This situation is helping to distinguish the respective value of “psychological treatments” as an umbrella term for many different psychosocial interventions and “psychotherapy” as a more specific non-manualized practice for dealing with the complexity of experience and behavior. Our stance is that both should coexist and even in blended treatments could be simultaneously harnessed in the same situation. Accordingly, it is essential to acknowledge that there are nuances that psychotherapy permits, and at least for now, the optimal way of delivering psychotherapy is in a shared physical space. However, VCP will definitely be expanded and hopefully integrated as a modality through which complex psychotherapeutic interventions can be delivered.

## Author Contributions

JF-Á drafted the manuscript and HF-Á provided critical revisions. Both authors reviewed and edited the final version of the manuscript.

### Conflict of Interest

The authors declare that the research was conducted in the absence of any commercial or financial relationships that could be construed as a potential conflict of interest.
